# Supplementary material for: Planar cell polarity signalling coordinates heart tube remodelling through tissue-scale polarisation of actomyosin activity
Source: Nat Commun. 2018 Jun 4;9:2161. doi: 10.1038/s41467-018-04566-1 (PMC5986786; doi:10.1038/s41467-018-04566-1)
Supplement: Supplementary file 1 — Supplementary Information [file 41467_2018_4566_MOESM1_ESM.pdf]

Planar Cell Polarity signalling coordinates heart tube remodelling through tissue-scale polarisation of actomyosin activity

Merks et al.

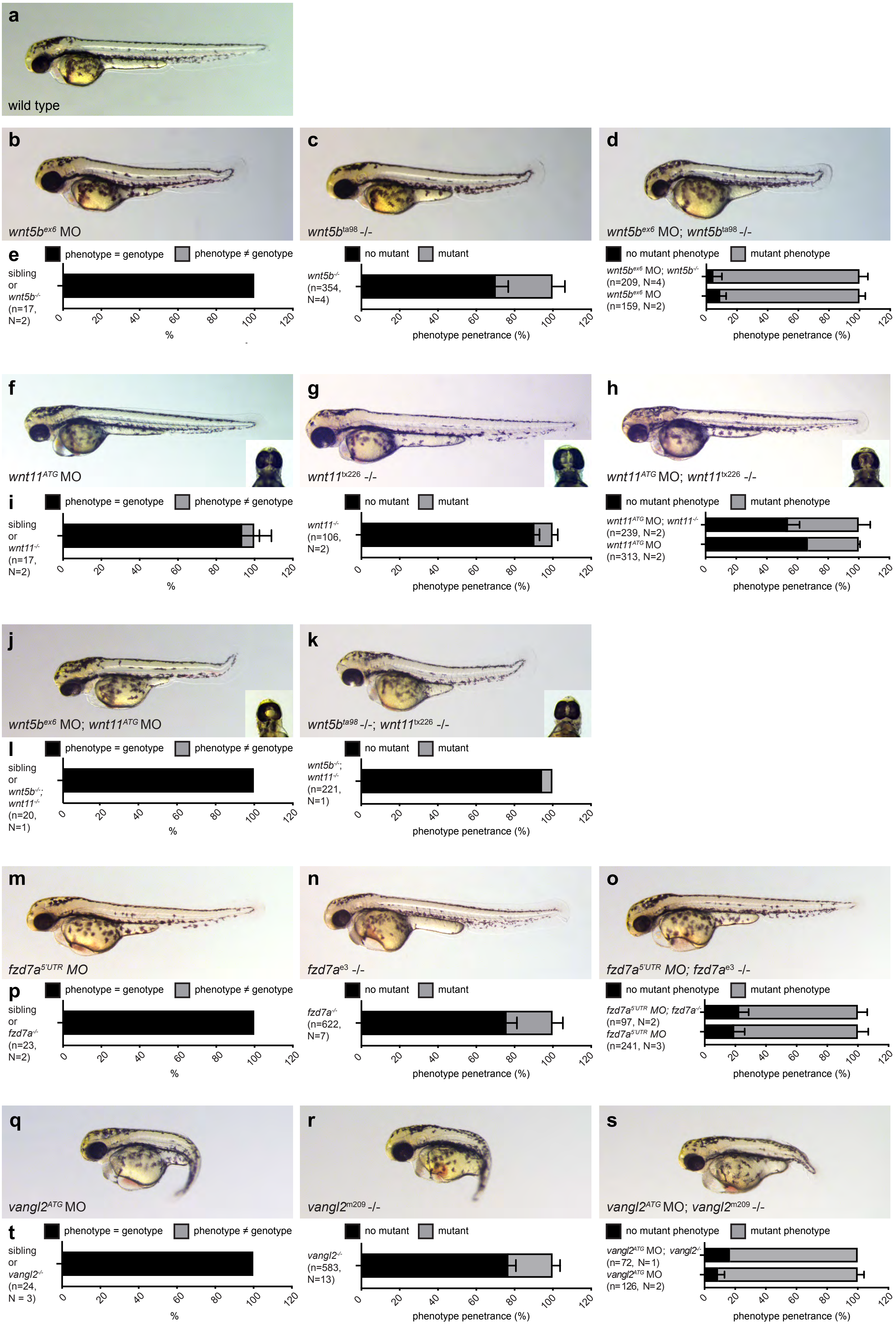

a
wild type
b
wnt5b^ex6 MO
c
wnt5b^ta98 -/-
d
wnt5b^ex6 MO; wnt5b^ta98 -/-
e
phenotype = genotype
phenotype ≠ genotype
sibling or wnt5b-/- (n=17, N=2)
%
no mutant
mutant
wnt5b-/- (n=354, N=4)
phenotype penetrance (%)
no mutant phenotype
mutant phenotype
wnt5b^ex6 MO; wnt5b-/- (n=209, N=4)
wnt5b^ex6 MO (n=159, N=2)
f
wnt11^ATG MO
g
wnt11^tx226 -/-
h
wnt11^ATG MO; wnt11^tx226 -/-
i
sibling or wnt11-/- (n=17, N=2)
wnt11-/- (n=106, N=2)
wnt11^ATG MO; wnt11-/- (n=239, N=2)
wnt11^ATG MO (n=313, N=2)
j
wnt5b^ex6 MO; wnt11^ATG MO
k
wnt5b^ta98 -/-; wnt11^tx226 -/-
l
sibling or wnt5b-/-; wnt11-/- (n=20, N=1)
wnt5b-/-; wnt11-/- (n=221, N=1)
m
fzd7a^5'UTR MO
n
fzd7a^e3 -/-
o
fzd7a^5'UTR MO; fzd7a^e3 -/-
p
sibling or fzd7a-/- (n=23, N=2)
fzd7a-/- (n=622, N=7)
fzd7a^5'UTR MO; fzd7a-/- (n=97, N=2)
fzd7a^5'UTR MO (n=241, N=3)
q
vangl2^ATG MO
r
vangl2^m209 -/-
s
vangl2^ATG MO; vangl2^m209 -/-
t
sibling or vangl2-/- (n=24, N = 3)
vangl2-/- (n=583, N=13)
vangl2^ATG MO; vangl2-/- (n=72, N=1)
vangl2^ATG MO (n=126, N=2)
0 20 40 60 80 100 120

Supplementary Figure 1: MO-induced phenotypes recapitulate PCP-mutants at 54 hpf

(**a**, **b**–**d**, **f**–**h**, **j**, **k**, **m**–**o**, **q**–**s**) Embryos at 54 hpf. Phenotypic and genotypic characterization of *wnt5b^{ex6}* morphants (**b**) and *wnt5b^{ta98}* mutants (**c**), *wnt11*^{ATG} morphants (**f**) and *wnt11*^{tx226} mutants (**g**), *wnt5b*^{ex6}; *wnt11*^{ATG} double morphants (**j**) and *wnt5b*^{ta98}; *wnt11*^{tx226} double mutants (**k**), *fzd7a*$^{5'UTR}$ morphants (**m**) and *fzd7a*^{e3} mutants (**n**) and *vangl2*^{ATG} morphants (**q**) and *vangl2*^{m209} mutants (**r**) compared to wild type embryos (**a**). Blinding experiments reveal 100% consistency of the phenotype (chosen and encrypted by person 1) with the genotype (carried out by person 2) for *wnt5b*^{ta98} mutants (**e**, left), *wnt5b*^{ta98}; *wnt11*^{tx226} double mutants (**l**, left), *fzd7a*^{e3} mutants (**p**, left) and *vangl2*^{m209} mutants (**t**, left); and 94% consistency for *wnt11*^{tx226} mutants (**i**, left) from a blinded mix of siblings and mutants. The penetrance of the respective mutant phenotypes is recorded with 30% for *wnt5b*^{ta98} mutants (**e**, middle), 10% for *wnt11*^{tx226} mutants (**i**, middle), 5% for *wnt5b*^{ta98}; *wnt11*^{tx226} double mutants (**l**, middle), 24% for *fzd7a*^{e3} mutants (**p**, middle) and 23% for *vangl2*^{m209} mutants (**t**, middle). To prove usage of the optimal dose of MO that does not lead to off-target effects the phenotype of individual MO-knockdowns was compared to MO-injections in the mutants: *wnt5b^{ex6}* morphants (91% phenotype) with *wnt5b^{ex6}* MO in *wnt5b*^{ta98} mutants (**d**, 95% phenotype); *wnt11*^{ATG} morphants (33% phenotype) with *wnt11*^{ATG} MO in *wnt11*^{tx226} mutants (**h**; 46% phenotype); *fzd7a*$^{5'UTR}$ morphants (81% phenotype) with *fzd7a*$^{5'UTR}$ MO in *fzd7a*^{e3} mutants (**o**; 77% phenotype) and *vangl2*^{ATG} morphants (91% phenotype) with *vangl2*^{ATG} MO in *vangl2*^{m209} mutants (**s**; 83% phenotype). Means±s.d.. Ordinary two-way ANOVA. Reported *P* values are multiplicity adjusted for each comparison. Uncapitalized n represents number of embryos.

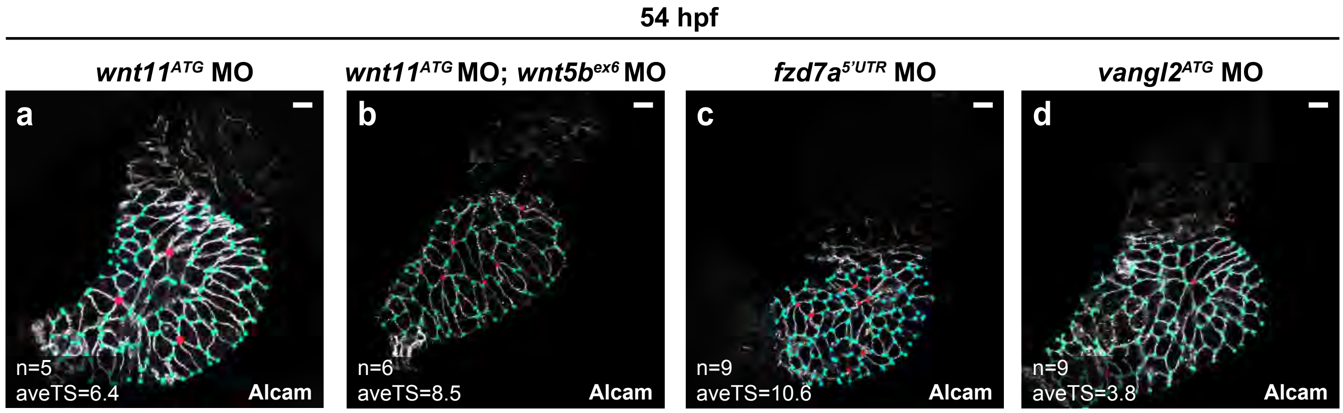

Supplementary Figure 2. Effects of reduction in the levels of non-canonical ligands Wnt11 and Wnt5b, and PCP core components on epithelial remodelling during chamber formation

(**a**–**d**) Top-down 0.5-µm confocal sections of hearts at 54 hpf stained for Alcam with transition states marked in red and 3-point vertices in blue. *Wnt11* deficiency slightly increases number of transition states (**a**) (compare to wt heart in **Fig. 2b**), while reduction in levels of both *wnt11* and *wnt5b* significantly increases the number of transition states (**b**). Hearts of *fzd7a*$^{5'UTR}$ morphants (**c**) display significantly more transition states than wild type, while hearts of *vangl2*^{ATG} morphants (**d**) show mild reduction in the number of transition states compared to wild type hearts. Scale bars, 10 µm. aveTS, average number of transition states.

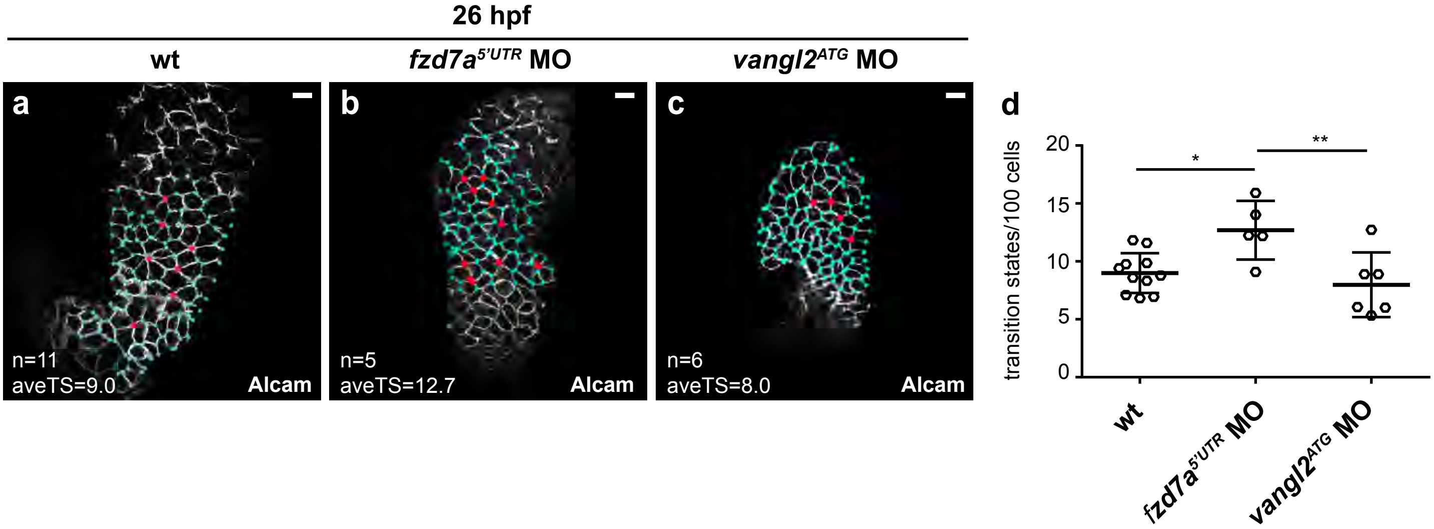

Supplementary Figure 3. Reduction in fzd7a results in the increased number of transition states already at LHT stage (**a**–**c**) Top-down 0.5-µm confocal sections of wild type (wt) (**a**), *fzd7a*- (**b**), and *vangl2*-deficient (**c**) hearts at 26 hpf stained for Alcam with transition states marked in red and 3-point vertices in blue. (**d**) Quantification at LHT reveals a significant increase of transition states in *fzd7a*$^{5'UTR}$ morphants (mean of 12.7 transitions per 100 cells, n=5) in comparison to wt hearts (mean of 9.0 transitions per 100 cells, n=1 1, *P=0.0184). Reduction of *vangl2* causes a slight decrease in the number of transitions compared to wt (mean of 8.0 transitions per 100 cells, n=6) *fzd7a*$^{5'UTR}$ MO vs. *vangl2*^{ATG} MO, **P=0.0072. Means±s.d.. Ordinary one-way ANOVA with Bonferroni's multiple comparison test. Reported P values are multiplicity adjusted for each comparison. Scale bars, 10 µm. aveTS, average number of transition states.

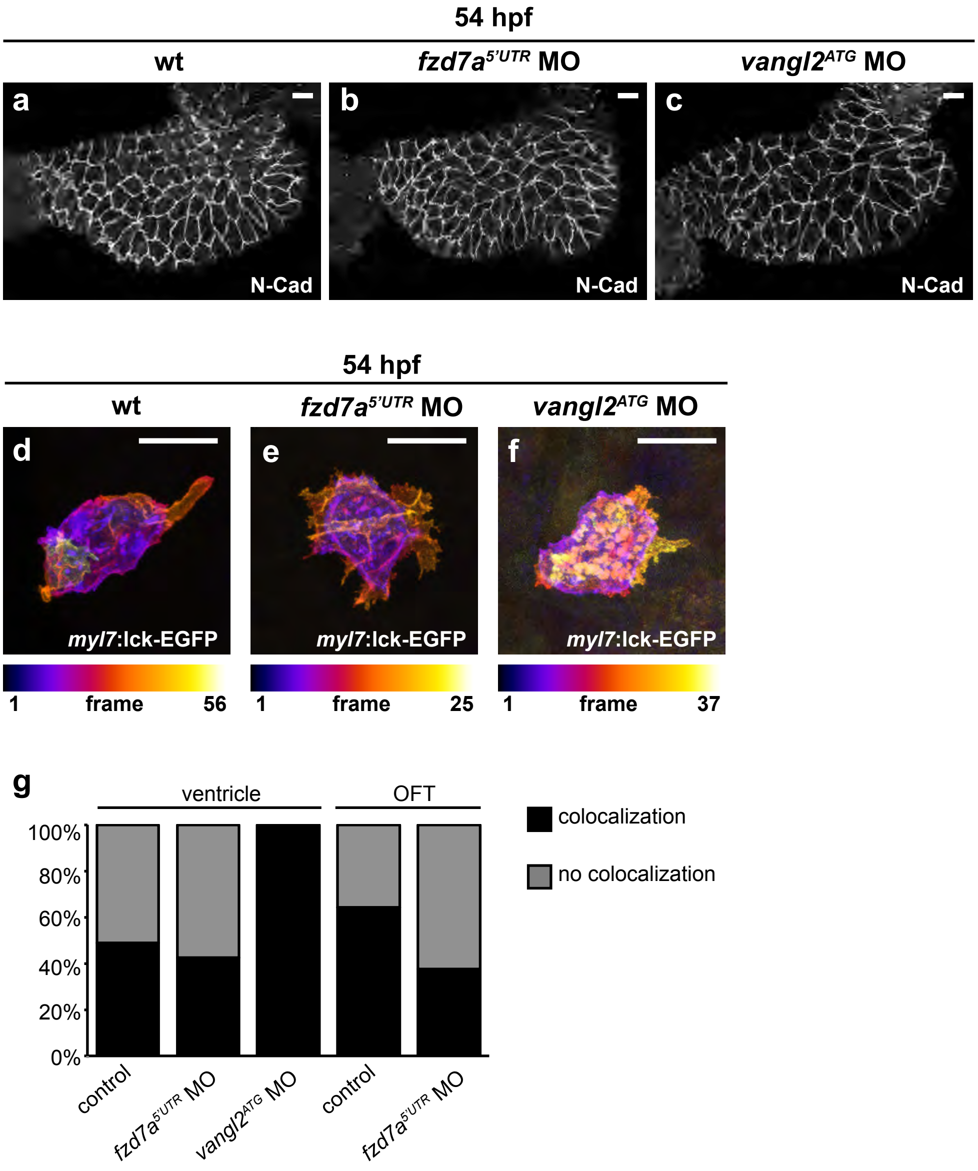

Supplementary Figure 4. PCP affects actomyosin contractile network

(**a**–**c**) Top-down 1-µm confocal Z-projection of 54 hpf hearts stained for N-Cadherin (N-Cad). In wild type (wt) hearts (n=15), N-Cad localizes uniformly to basolateral cell membranes (**a**). In both *fzd7a*- (n=9) and *vangl2*-deficient hearts (n=9), N-Cad localization and abundance were unchanged (**b**, **c**). (**d**–**f**) Maximal confocal depth Z-projection (number of frames given in the pictures, z-stack size 0.5 µm) of single cells expressing membrane-associated EGFP (transient expression of *myl7*:lck-EGFP), depicting apical parts in blue/magenta and basal parts in white/yellow. At 54 hpf, wt cardiomyocytes show few basal cell protrusions (**d**). Cardiomyocytes of *fzd7a*$^{5'UTR}$-morphant hearts are apically constricted, forming numerous pronounced basal cell protrusions (**e**). Reduced levels of *vangl2* mildly affect cardiomyocyte shape with overall slightly smoother surfaces and distorted basal membrane (**f**). (**g**) Graph displaying percentage of F-Actin-pMRLC co-localisation at transitions in the ventricle and outflow tract (OFT) in stacked bar graphs. In control hearts, 49% of F-Actin and pMRLC co-localize at the ventricular transitions (20/41 ventricular transitions from 10 hearts), in the OFT of control hearts there is 64% co-localisation (9/14 OFT transitions from 9 hearts). Loss of *fzd7a* just slightly changes F-Actin-pMRLC co-localisation in the ventricle (43% co-localisation, 17/40 ventricular transitions from 9 hearts). More prominent region-specific difference is seen in co-localisation in the OFT (38% co-localisation, 3/8 OFT transitions from 6 hearts). *Vangl2* deficiency leads to 100% co-localisation of F-Actin and pMRLC in the ventricular transitions (21/21 ventricular transitions from 7 hearts), no transitions are observed in OFT. OFT, outflow tract.

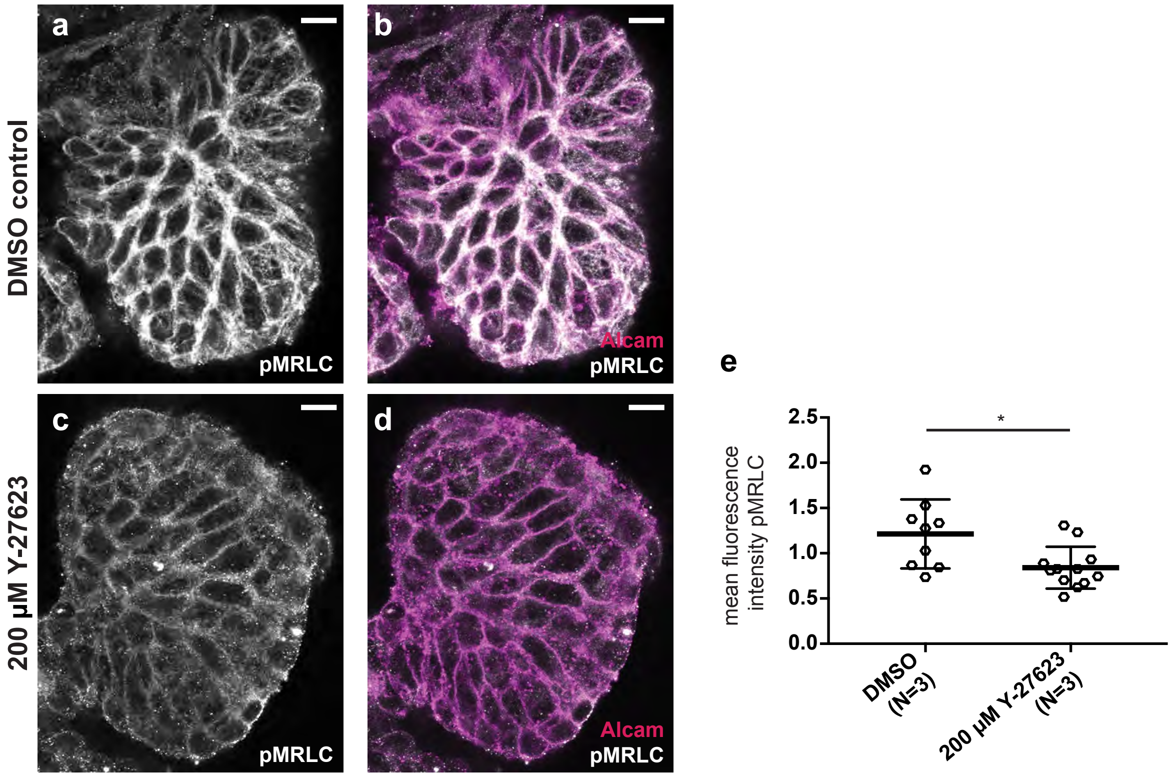

Supplementary Figure 5. Phosphorylated MRLC is significantly reduced upon treatment with ROCK inhibitor Y-27623

(**a**–**d**) Top-down 0.5-µm confocal sections of ventricles at 54 hpf of DMSO treated (**a**, **b**) and with ROCK inhibitor treated hearts (**c**, **d**) at 54 hpf stained for pMRLC (**a**–**d**) and Alcam (magenta in **b**, **d**). (**e**) Quantification of ventricular mean fluorescence intensities of pMRLC reveals the reduction of levels of pMRLC after treatment with 200 µM ROCK inhibitor Y-27632 dihydrochloride (n=12, N=3) compared to DMSO control (n=9, N=3). Means±s.d.. *P=0.023, unpaired t-test with Welch correction. Scale bars, 10 µm. Uncapitalized n represents number of hearts.

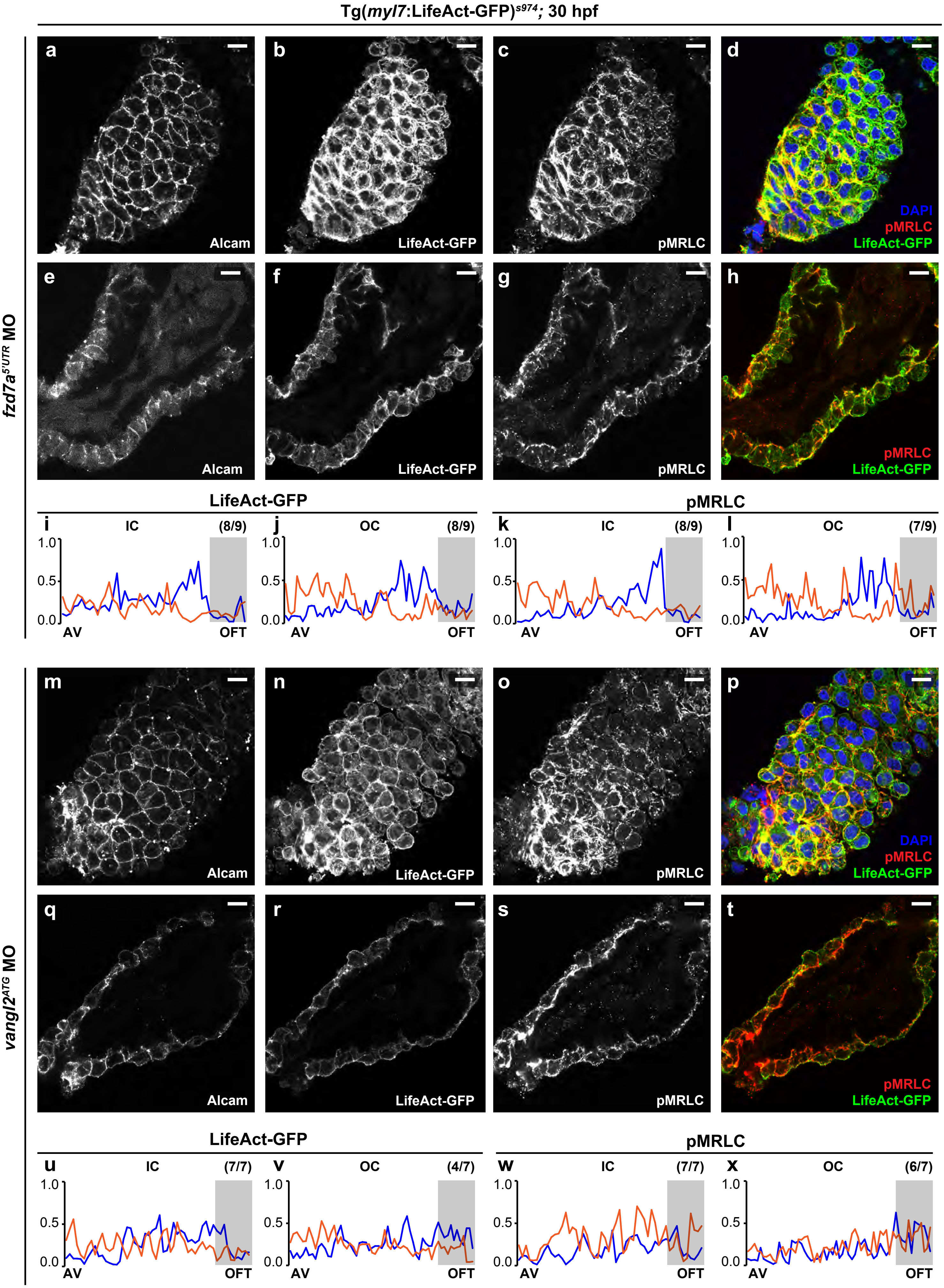

Tg(myl7:LifeAct-GFP)s974; 30 hpf
fzd7a5'UTR MO
a
Alcam
b
LifeAct-GFP
c
pMRLC
d
DAPI
pMRLC
LifeAct-GFP
e
Alcam
f
LifeAct-GFP
g
pMRLC
h
pMRLC
LifeAct-GFP
LifeAct-GFP
pMRLC
i
IC
(8/9)
j
OC
(8/9)
k
IC
(8/9)
l
OC
(7/9)
1.0
0.5
0.0
AV
OFT
vangl2ATG MO
m
Alcam
n
LifeAct-GFP
o
pMRLC
p
DAPI
pMRLC
LifeAct-GFP
q
Alcam
r
LifeAct-GFP
s
pMRLC
t
pMRLC
LifeAct-GFP
LifeAct-GFP
pMRLC
u
IC
(7/7)
v
OC
(4/7)
w
IC
(7/7)
x
OC
(6/7)

Supplementary Figure 6: Loss of planar-polarized actomyosin in the absence of *fzd7a* and *vangl2*

(**a**–**d**, **m**–**p**) Top-down 1-µm confocal section of the *fzd7a*$^{5'UTR}$ (**a**–**d**) and *vangl2*^{ATG} (**m**–**p**) morphant hearts at 30 hpf. Both F-Actin (*myl7*:LifeAct-GFP, counter-stained with anti-GFP) (**b**, **n**, green in **d**, **p**) and phospho-MRLC (**c**, **o** red in **d**, **p**) are localised throughout the ventricle. (**e**–**h**, **q**–**t**) Mid-sagittal 1-µm confocal section through the *fzd7a*$^{5'UTR}$ (**e**–**h**) and *vangl2*^{ATG} (**q**–**t**) morphant hearts at 30 hpf. F-actin (*myl7*:LifeAct-EGFP, counter-stained with anti-GFP) (**f**, **r**, green in **h**, **t**) and pMRLC (**g**, **s**, red in **h**, **t**) showing randomized localization throughout the ventricle with clear basal localization in the OFT. Hearts were counter-stained for Alcam to visualize membranes (**a**, **e**, **m**, **q**), and DAPI to label nuclei (blue in **d**, **p**), respectively. Scale bars, 10 µm. (**i**–**l**, **u**–**x**) Line plot profiles of apical (blue) and basal (orange) membranes in IC and OC revealing the absence of apical accumulation of the actomyosin from basal in AV/proximal ventricle to apical in distal ventricle/OFT in loss of both *fzd7a* (**i**–**l**) and *vangl2* (**u**–**x**). OFT region is highlighted by a grey rectangle. Numbers given in the line plot profiles represent the number of hearts showing the absence of apical accumulation of actomyosin out of total number of hearts. IC, inner curvature. OC, outer curvature.

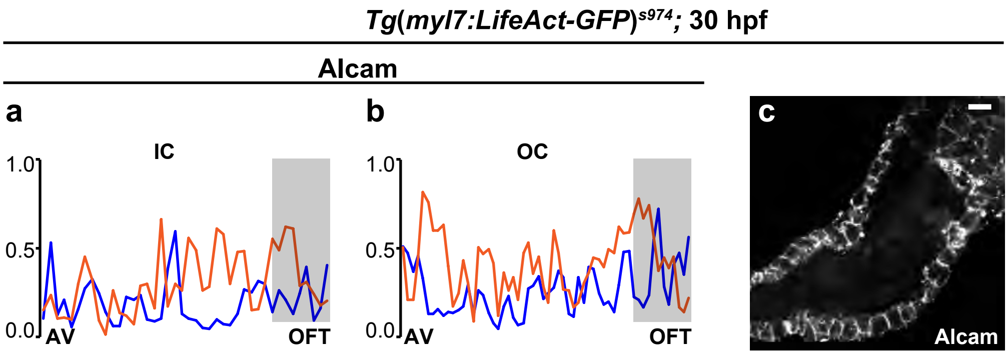

Supplementary Figure 7. Localization of the membrane marker Alcam is not polarized throughout the ventricle
(**a**, **b**) Line plot profiles of apical (blue) and basal (orange) membranes in IC and OC display an equal distribution of Alcam across the ventricle. (**c**) Mid-sagittal 1-μm confocal section through the linear heart tube at 30 hpf stained for Alcam. Scale bar, 10 μm. IC, inner curvature. OC, outer curvature.

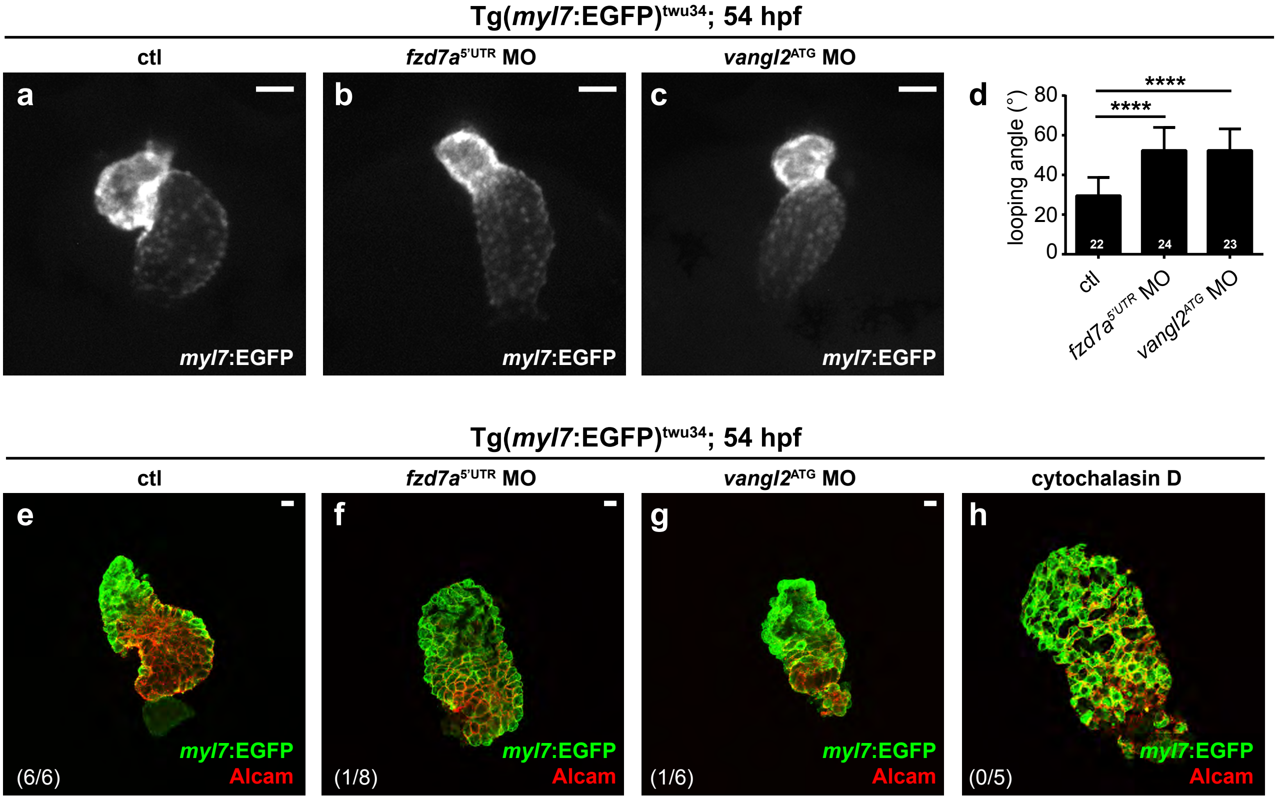

Supplementary Figure 8. Cardiac looping fails in fzd7a$^{5'UTR}$ and vangl2^{ATG} morphant hearts

(**a**–**c**) Cardiac looping of Tg(*myl7*:EGFP)^{tw34} embryos at 54 hpf. Scale bars, 50 μm. Cardiac looping in *fzd7a*- (**b**) and *vangl2*- (**c**) deficient hearts is impaired as compared to control (**a**) hearts. (**d**) Quantification of cardiac looping angle, defined as an angle between the plane of atrio-ventricular junction and the embryo midline axis. *fzd7a*$^{5'UTR}$ (**b**) and *vangl2*^{ATG} morphant hearts (**c**) display significantly greater average looping angle than control. Means±s.d.. ****P<0.0001, ordinary one-way ANOVA with Bonferroni's multiple comparison test. Reported P values are multiplicity adjusted for each comparison. (**e**–**g**) Cardiac looping of explanted hearts from Tg(*myl7*:EGFP)^{tw34} embryos. At 28 hpf linear heart tubes were dissected from control (**e**) or *fzd7a*$^{5'UTR}$ (**f**) and *vangl2*^{ATG} morphant (**g**) embryos, incubated in supplemented tissue culture medium for 24 h prior to fixation and imaged. Treatment of explanted control hearts with 10 μM cytochalasin D during ex vivo culture for 24 h inhibits heart looping (**h**), similar to the loss of *fzd7a* and *vangl2*. Numbers given in the panels **e**–**g** represent the numbers of looped hearts out of total number of hearts. N=1. Scale bars, 10 μm
