# Supplementary material for: Planar cell polarity signalling coordinates heart tube remodelling through tissue-scale polarisation of actomyosin activity
Source: Nat Commun. 2018 Jun 4;9:2161. doi: 10.1038/s41467-018-04566-1 (PMC5986786; doi:10.1038/s41467-018-04566-1)
Supplement: Supplementary file 3 — Description of Additional Supplementary Files [file 41467_2018_4566_MOESM3_ESM.docx]

**Description of Additional Supplementary Files**

File Name: Supplementary Movie 1

Description: Resolving transition state during critical time of cardiac chamber formation. Movie follows a resolving transition state in a non-contractile *tnnt2a^ATG^* morphant heart expressing membrane-associated EGFP under *myl7* promoter (Tg(*myl7*:lckEGFP)^md71^). At 26 hpf, the LHT displays a transition state consisting of five cardiomyocytes that share a common cell boundary (arrow). During the following 5 h this transition state resolves fully and at 31 hpf a new cell junction is formed (arrowhead). 5 fps. Scale bar, 10 μm.

File Name: Supplementary Software 1

Description: Custom MatLab script ‘Zf heart cell orientation.rar’.
